# Cortisol awakening response in pregnant women with depressive disorders: a potential marker of recovery status from pregnancy to postpartum

**DOI:** 10.1016/j.cpnec.2025.100297

**Published:** 2025-04-28

**Authors:** Carlinde W. Broeks, Babette Bais, Rien Van, Hilmar H. Bijma, Elisabeth F.C. van Rossum, Witte J.G. Hoogendijk, Mijke P. Lambregtse-Van den Berg, Astrid M. Kamperman

**Affiliations:** a Department of Psychiatry, Erasmus MC, University Medical Center Rotterdam, Rotterdam, the Netherlands; b Department of Child and Adolescent Psychiatry/Psychology, Sophia Children's Hospital Erasmus MC, University Medical Center Rotterdam, Rotterdam, the Netherlands; c Arkin Institute for Mental Health, Amsterdam, the Netherlands; d Epidemiological and Social Psychiatric Research Institute, Erasmus MC, University Medical Center Rotterdam, Rotterdam, the Netherlands; e Department of Obstetrics and Gynaecology, Division of Obstetrics and Fetal Medicine, Erasmus University Medical Centre Rotterdam, Rotterdam, the Netherlands; f Department of Care Ethics, University of Humanistic Studies, Utrecht, the Netherlands; g Department of Internal Medicine, Division of Endocrinology, Erasmus University Medical Center Rotterdam, and Obesity Center CGG, Rotterdam, the Netherlands

**Keywords:** Peripartum depression, Cortisol awakening response (CAR), Postpartum depression predictors, Hypothalamic-pituitary-adrenal (HPA) axis, Salivary cortisol levels

## Abstract

**Introduction:**

Dysregulation of the hypothalamic-pituitary-adrenal (HPA) axis has been linked to peripartum depression, potentially contributing to symptom persistence. This study examines the relationship between the cortisol awakening response (CAR) during pregnancy and depressive symptom reduction postpartum.

**Methods:**

Pregnant women with a current depressive episode were included in this study, part of a larger RCT on bright light therapy. At baseline (12–32 weeks of pregnancy), participants provided saliva samples at awakening, +30, and +60 min post-awakening. The CAR was assessed using three measures: area under the curve relative to ground (AUCg), area under the curve relative to increase (AUCi), and peak reactivity (difference between awakening and +30 min cortisol levels). Depressive symptoms were measured using the Hamilton Depression Rating Scale (HAM-D) at baseline and two months postpartum. Linear regression models assessed associations between CAR measures and depressive symptom change, adjusting for relevant covariates.

**Results:**

The study included 55 pregnant women (mean age: 32.3 years, SD 4.8; mean gestational age: 19.7 weeks). Mean HAM-D scores decreased from 16.7 (SD 5.3) at baseline to 5.7 (SD 5.6) postpartum. Higher AUCi during pregnancy was associated with less symptom reduction postpartum (unadjusted β = 0.36, p = .02; adjusted β = 0.36, p = .02), as was peak cortisol reactivity (unadjusted β = 0.33, p = .03; adjusted β = 0.32, p = .03), while AUCg showed no significant association with symptom change (unadjusted p = .19; adjusted p = .28).

**Conclusion:**

Higher AUCi and peak cortisol reactivity of the cortisol awakening response during pregnancy were linked to persistence of depressive symptoms postpartum, suggesting that heightened cortisol reactivity to awakening may indicate persistent stress vulnerability in peripartum depression. Total cortisol output (AUCg) was not predictive of recovery. These findings underscore the potential relevance of stress reactivity over basal cortisol levels in peripartum depression and highlight the need for further research in larger samples to elucidate the usefulness in clinical practice.

## Highlights

• *Heightened cortisol awakening response (CAR) during pregnancy predicts less symptom improvement postpartum.*
• *AUCi and peak reactivity, but not total cortisol output (AUCg), were associated with persistent depressive symptoms.*
• *Findings suggest that stress reactivity, rather than basal cortisol levels, may play a key role in peripartum depression.*
• *CAR may serve as a potential biomarker for identifying women at risk of prolonged postpartum depression.*

## 1 Introduction

Depressive disorders during pregnancy and postpartum pose significant risks for both mother and child, affecting maternal well-being, child outcomes, and early mother-infant interactions [1]. Emerging evidence implicates the endocrine system, specifically the hypothalamic–pituitary–adrenal (HPA) axis, in the onset and persistence of depressive disorders during pregnancy and postpartum [2]. Dysregulation of the HPA axis, including basal cortisol levels and stress reactivity, has been associated with both active and remitted depression [3,4].

During pregnancy, the maternal hypothalamic-pituitary-adrenal (HPA) axis undergoes significant adaptations, leading to increased circulating cortisol levels while simultaneously showing signs of blunted negative feedback regulation [5]. These changes are driven by rising levels of placental corticotropin-releasing hormone (CRH), increased corticosteroid-binding globulin (CBG), and alterations in CRH-binding protein (CRH-BP), which modulate CRH bioavailability throughout gestation, leading to a two-to threefold rise in maternal plasma cortisol [6,7]. Given that elevated and decreased cortisol levels have been associated with depression in non-pregnant populations, it has been hypothesized that these pregnancy-related hormonal changes may contribute to depression.

However, not all women with peripartum depression show clear HPA axis dysregulation, which can manifest at different levels. Some individuals exhibit elevated cortisol levels due to increased HPA axis activation, while others show impaired regulatory feedback, leading to altered cortisol reactivity rather than consistently high levels [8,9]. Importantly, not all women with peripartum depression display clear markers of HPA axis dysfunction, highlighting the complexity of its role in perinatal mental health.

A potential marker of stress regulation is the Cortisol Awakening Response (CAR), which represents the natural rise in cortisol levels within 20–30 min after awakening. This increase in cortisol upon waking is thought to prepare the body for the anticipated demands of the day, making it a key indicator of the body's ability to mobilize energy and cope with stress [10]. The CAR reflects HPA axis reactivity and has been widely studied in stress-related disorders, including depression [11]. Clinically, CAR has been proposed as a biomarker of an individual's ability to regulate stress and adapt to challenges [5,12,13]. The CAR is generally considered a stable and reliable measure in healthy populations, with several studies demonstrating its stability over short- and long-term intervals [14]. Recent evidence from Wang et al. (2024) indicates that depressed individuals tend to exhibit an elevated CAR, with sex-specific differences showing higher CAR responses in depressed females compared to males [11].

In healthy pregnant women, the overall circadian rhythm and awakening response of cortisol are preserved [15,16], although the CAR might decrease in later pregnancy [17,18]. Several studies have evaluated the relationship between CAR and peripartum depression, suggesting a potential role for CAR in understanding HPA-axis alterations during this period. Some research has reported that pregnant women with major depressive disorder (MDD) exhibit lower awakening cortisol levels [[19], [20], [21]], while others have found an increased CAR in this population [22,23]. Although some studies have not observed significant differences between depressed and non-depressed pregnant women [24,25], such variability may reflect methodological differences or sample characteristics. Despite this variability in findings, CAR appears to be a valuable biomarker for investigating peripartum depression and its underlying biological mechanisms.

Given the mixed findings regarding the relationship between the CAR and peripartum depression, there remains a critical need to clarify how CAR during pregnancy correlates with depressive symptoms. Identifying a biological marker that can be used as a marker to predict the course of symptoms and guide treatment for peripartum depression would offer significant clinical value. This study examines the relationship between the CAR during pregnancy, before treatment, and depressive symptoms postpartum. The sample consists of pregnant women with depressive disorder from a randomized controlled trial (RCT) on bright light therapy [26]. While both treatment and placebo groups showed significant symptom reduction - suggesting spontaneous remission, placebo effects, or behavioral changes - our study focuses on CAR as a potential marker for symptom change.

We hypothesize that in women with depression during pregnancy, a more pronounced CAR - characterized by a steeper increase and/or higher peak in cortisol levels after awakening - may be related to a smaller reduction in depressive symptoms after delivery. Elevated CAR has been linked to heightened stress reactivity and persistent depressive symptoms in other populations [[27], [28], [29]]. However, this relationship remains understudied in pregnancy, where hypercortisolism and HPA axis adaptations may further modulate CAR dynamics. We therefore explore how specific CAR measures – area under the curve with respect to ground (AUCg) and increase (AUCi), and peak reactivity - relate to symptom change in pregnant women.

Given the impact of peripartum depression on maternal and infant health, identifying potential biomarkers like CAR could support early intervention strategies. Hormonal fluctuations play a key role in peripartum depression [30,31], and studies suggest CAR variations may indicate depression risk [28,29]. This study employs longitudinal cortisol assessments to explore the potential role of CAR in peripartum mental health.

## 2 Materials and methods

### 2.1 Study design

This study was part of a randomized controlled trial (RCT), in which pregnant women were randomly allocated to treatment with either bright light therapy (BLT) or a placebo condition with dim red light therapy (DRLT) during 6 weeks [26,32]. The original sample consisted of 67 women diagnosed with a depressive disorder during pregnancy.

Although different studies in small samples found positive effects of BLT on antepartum depression [[33], [34], [35], [36]], our RCT showed no statistically significant differences in symptom change scores between BLT and DRLT. Symptoms significantly improved in both treatment arms, with HDRS scores decreasing from a mean of 16.7 (SD 5.3) to 5.7 (SD 5.6) two months postpartum, corresponding to an average symptom reduction of 65.7 %.

The Medical Ethical Committee of the Erasmus Medical Center, Rotterdam, The Netherlands, approved this research project (MEC-2015–731).

### 2.2 Participants

Participants were included between week 12 and 32 of pregnancy (gestational age confirmed by first-trimester ultrasound). Diagnosis of a depressive disorder was established through a structured clinical interview for DSM disorders (SCID) conducted by a trained assessor, following the Diagnostic and Statistical Manual of Mental Disorders. Recruitment took place in the Netherlands from 2016 to 2019. Details on recruitment and in- and exclusion criteria are outlined elsewhere [26].

For the current analysis, additional exclusion criteria included the use of locally administered or systemic corticosteroids and obstetric complications (e.g. preterm birth) (n = 0), as these factors could be related to cortisol levels. However, no participants in the study experienced preterm birth (n = 0), so this criterion did not lead to any exclusions. Out of the original 67 women included in the study, 10 were excluded due to missing cortisol measurements and 2 were excluded for corticosteroid use, leaving 55 women in the final sample.

At baseline, socio-demographic information was collected, including age, country of birth, educational level, marital status, and body mass index (BMI). Obstetric details such as gestational age, whether the pregnancy was planned, and parity were also recorded. Psychiatric information was gathered through assessments of substance use (smoking, alcohol, drugs), current and past medication use, present depressive symptoms, psychiatric history, and somatic conditions. Participants were screened for depressive disorders and potential comorbidities (e.g., generalized anxiety disorder, panic disorder) using the SCID. The SCID was also used to assess prior depressive episodes. To verify medication use and confirm that participants met the inclusion criteria, the general practitioner was contacted.

### 2.3 Measures

#### 2.3.1 Dependent variable

##### 2.3.1.1 Hamilton rating scale for depression (HAM-D)

The Hamilton Rating Scale for Depression (HAM-D) is a clinician-administered tool for assessing the severity of depression. The 17-item version of the HAM-D evaluates a range of depressive symptoms, including mood, sleep disturbances, anxiety, and somatic complaints. Each item is scored on a scale of 0–2 or 0 to 4, with total scores ranging from 0 to 52, where higher scores indicate more severe depression.

Although the HAM-D was measured at multiple time points during pregnancy and postpartum in the original study, we selected baseline and two months postpartum for this analysis to capture changes over a longer timeframe encompassing the full peripartum period. These time points also coincided with cortisol sampling, enabling a direct comparison between CAR and changes in depressive symptoms.

#### 2.3.2 Primary independent variables

##### 2.3.2.1 Cortisol Awakening Response (CAR)

The Cortisol Awakening Response (CAR) reflects the body's ability to regulate stress and adapt to daily challenges. Upon awakening, cortisol levels typically show a rapid increase, peaking around 30–45 min post-awakening, before gradually declining. This surge in cortisol is thought to prepare the body for the demands of the day and is influenced by various factors, including sleep quality, psychological stress, and overall HPA-axis function.

In this study, salivary cortisol was measured at three key time points: upon waking (T1), 30 min post-awakening (T2), and 60 min post-awakening (T3). These time points are widely recognized as the most valid for capturing the dynamics of the CAR. To ensure accurate measurements, participants were instructed to refrain from food, drink, toothbrushing, or smoking for 1 h before sampling. Cortisol samples were collected using Salimetric Swabs (Salimetrics, State College, PA) and centrifuged for 10 min at ∼1600×*g* (equivalent to 3000 rpm in our system) to extract saliva, which was subsequently frozen at −80 °C. Cortisol concentrations were measured using liquid chromatography–tandem mass spectrometry (LC-MS/MS) on a Waters XEVO-TQ-S system (Waters Corporation, Milford, MA, USA). Outlier detection was performed at all three time points (values > 3 SD above or below the mean), but no outliers were identified (n = 0). To approximate a normal distribution, cortisol measurements were log-transformed (10log).

To quantify the CAR, we calculated the area under the curve (AUC) following the approach recommended by Pruessner et al. [37]. The AUC with respect to increase (AUCi) captures the dynamic rise in cortisol over time, providing a measure of HPA-axis reactivity to awakening. The AUC with respect to ground (AUCg) reflects overall cortisol secretion during the awakening period, offering a broader index of cortisol output. Additionally, peak reactivity - defined as the absolute change in cortisol from waking (T1) to its peak at 30 min post-awakening (T2) - was assessed as a marker of cortisol responsiveness. These indices of the CAR were used as independent variables to explore their association with changes in depressive symptoms from baseline to postpartum.

#### 2.3.3 Potential confounders

Salivary cortisol measurements in pregnant women can be influenced by several potential confounders that may affect their accuracy and interpretation. To account for these factors, a propensity score correction was applied, incorporating key baseline characteristics associated with cortisol regulation. Below, we outline the main confounders and how they were handled in the analyses.

One factor is gestational age, as cortisol levels naturally increase as pregnancy progresses, particularly in the third trimester [38]. BMI was also considered, given its association with HPA-axis function and potential influence on cortisol secretion [39]. Medication use is another potential confounder. Regarding psychotropic medication, antidepressant use (n = 7) was documented, but no significant associations were found with any of the CAR indices. Participants using corticosteroids (n = 2) were excluded, as exogenous corticosteroids can suppress the HPA axis [40]. Nevertheless, medication use - including both psychotropic and somatic medications - was included in the propensity score.

Additionally, smoking status was considered, given its potential effect on cortisol metabolism [41], though only one participant reported smoking, making its impact negligible. Country of birth may intervene with the CAR [42]. Peripartum complications and underlying medical conditions such as thyroid dysfunction may also interfere with cortisol regulation, but no somatic issues of that nature were reported by the participants [43].

Another key factor is diurnal variation, given that cortisol follows a natural circadian rhythm, peaking shortly after awakening and gradually decreasing throughout the day. Ideally, exact awakening times should be recorded to precisely capture the CAR. However, in this study, awakening times were not directly recorded; instead, participants provided self-reported chronotype information (morning preference, evening preference, or neutral), which was used as a proxy to examine potential variations in cortisol secretion patterns.

As expected with successful randomization, no significant baseline differences were found in cortisol levels between treatment groups (awakening cortisol: p = .449, AUCi: p = .442, AUCg: p = .286, peak reactivity: p = .426). Nevertheless, treatment allocation (bright light therapy vs. placebo) was included in the propensity score to account for any potential residual effects on cortisol dynamics.

To adjust for these potential confounders while maintaining statistical power, a propensity score was calculated using a multiple linear regression model. The propensity score incorporated gestational age at baseline, pregnancy BMI, medication use (excluding corticosteroids due to direct HPA-axis suppression), country of birth, smoking status, chronotype, and treatment allocation. A sensitivity analysis confirmed that the inclusion of the propensity score did not alter the primary findings.

### 2.4 Analytic strategy

All statistical analyses were conducted using SPSS for Windows, version 29 (IBM, Chicago, Illinois). First, outlier detection was performed on cortisol values (≥3 SD above or below the mean per time point), but no outliers were identified (n = 0). Cortisol values were log-transformed (10log) to approximate a normal distribution.

To examine the association between the CAR and depressive symptom change from pregnancy to postpartum, we performed a series of linear regression analyses with change in Hamilton Depression Rating Scale (HAM-D) scores as the dependent variable. The independent variables were AUCi, AUCg, and peak reactivity [37].

To account for potential confounding, a propensity score correction was applied. The propensity score was estimated using a multiple linear regression model, predicting CAR based on key baseline characteristics as described above. The predicted values from this model were used as a single propensity score variable in subsequent regression analyses.

As a sensitivity analysis, we repeated the regression models using postpartum HAM-D scores as the outcome, with baseline HAM-D included as a covariate, alongside the propensity score, to reduce the risk of collider bias, ensuring that associations between CAR and depressive symptoms were not confounded by initial depression severity. Results remained consistent, with slightly stronger effects.

At baseline, HAM-D measurements were available for 55 participants, and at two months postpartum, for 43 participants. Missing data were handled using pairwise deletion.

## 3 Results

### 3.1 Background and clinical characteristics

Table 1 presents the demographic and clinical characteristics of the sample. The study included 55 pregnant women with a mean age of 32.3 years (SD 4.8) and a mean gestational age of 19.7 weeks (range: 12–32 weeks). The majority of participants (80 %) were native Dutch, while 9 % were of European and 11 % of non-European descent. Most participants were married or partnered (86.4 %), and a majority had a higher level of education (69.1 %). In terms of parity, 52.7 % were primiparous. Almost all participants (98.2 %) reported not smoking during pregnancy.

**Table 1:** Descriptive statistics of pregnant women (n = 55) from the *Bright-Up* study with availability of salivary cortisol measurements at baseline.

Participants (n = 55)	
*Demographic characteristics*	
Age, years (mean, SD)	32.3 (SD 4.8)
Gestational age, weeks (mean, range)	19.7 (12–32)
Country of birth: • Native Dutch • European/non-European	44 (80.0 %) 5/6 (9.0/11.0 %)
Married/partnered (y)	53 (96.4 %))
Education level (higher)	38 (69.1 %)
Parity (primiparous)	29 (52.7 %)
Maternal smoking (y)^a^	1 (1.8 %)
BMI^a^	26.1 (SD 5.3)
*Clinical characteristics*	
Psychiatric disorder • Current depressive disorder • Comorbid psychiatric disorder o Anxiety disorder^b^ o Obsessive-compulsive disorder o PTSD	55 (100 %) 30 (54.5 %) 11 (20.0 %) 8 (14.5 %)
Duration of depression (weeks) (mean, SD)	22.1 (SD 17.8)
Depressive episodes in the past (n = 46) • 0 • 1 • >1	19 (41.3 %) 20 (43.5 %) 7 (15.2 %)
Medication use • Antidepressants^c^	7 (12.7 %)
Hamilton Depression Rating Scale (HAM-D, 17 items) • Mean value at baseline (n = 55) • Mean values 2 months postpartum (n = 43) • Mean difference from baseline to postpartum (n = 43)	Mean, SD 16.7 (SD 5.3) 5.7 (SD 5.6) −11.1 (SD 6.3)

Note:

a At time of cortisol sampling, at baseline (12–32 weeks of pregnancy).

b Incl. panic disorder, agoraphobia, generalized anxiety disorder, specific anxiety disorder, social anxiety disorder.

c Only Selective Serotonin Reuptake Inhibitor (SSRI) were used.

Clinically, all participants (100 %) were diagnosed with a current depressive disorder, with 54.5 % also presenting comorbid psychiatric conditions, primarily anxiety disorders (20 %). The average duration of the current depressive episode was 22.1 weeks (SD 17.8), and 43.5 % had experienced one previous depressive episode. A small portion of participants (12.7 %) were using antidepressants. The mean Hamilton Depression Rating Scale (HAM-D) score at baseline was 16.7 (SD 5.3), which significantly reduced to 5.7 (SD 5.6) two months postpartum, showing a mean reduction of −11.1 points (SD 6.3). On average, participants scored around the threshold for moderate depression (HAM-D ≥17), with individual scores ranging from mild to severe depressive symptoms at baseline.

Before investigating the association between the Cortisol Awakening Response (CAR) and depressive symptom reduction, we first examined whether depressive symptom change differed between the treatment arms (bright light therapy vs. placebo). At baseline, the light therapy group had a mean HAM-D score of 15.7 (SD 5.4) compared to 17.8 (SD 5.1) in the placebo group (p = .15). Two months postpartum, the scores were 6.9 (SD 6.4) in the light therapy group and 4.2 (SD 4.2) in the placebo group (p = .11). As no significant differences were observed, we proceeded to examine biological predictors of symptom change, focusing on CAR as a potential biomarker.

### 3.2 Cortisol concentrations and Cortisol Awakening Response (CAR)

The mean cortisol concentration at awakening was 8.5 nmol/L (SD 3.6), which increased to 10.4 nmol/L (SD 4.6) 30 min after awakening, before returning to 8.5 nmol/L (SD 3.7) at 60 min post-awakening. The area under the curve with respect to the ground (AUCg), representing total cortisol output, had a mean value of 126.9 (SD 32.1) nmol/L × min. The area under the curve with respect to the increase (AUCi), a measure of cortisol reactivity, was 5.8 (SD 14.8). While these values provide a quantitative measure of cortisol secretion and response, direct clinical interpretation remains challenging, as standardized reference values for AUCg and AUCi in peripartum populations are lacking. Prior research has demonstrated substantial variability in these measures depending on study population, methodological differences, and individual stress response factors [44]. All values are presented in Table 2.

**Table 2:** Cortisol concentrations (mean, SD).

Cortisol concentrations (nmol/L) and Cortisol Awakening Response (CAR)	
Awakening	8.5 (SD 3.6)
Awakening + 30 min	10.4 (SD 4.6)
Awakening + 60 min	8.5 (SD 3.7)
AUCg^a^	126.9 (SD 32.1)
AUCi^a^	5.8 (SD 14.8)
Peak reactivity^a^	0.2 (SD 0.4)

Model 1		B	SE	β	p-value
Step 1	CAR - AUCi	0.14	0.06	0.36	0.02∗
Step 2	CAR - AUCi	0.15	0.06	0.36	0.03∗
	Propensity score	−0.10	0.08	−0.18	0.22

Note.

a Cortisol values were log-transformed before calculation of the AUCg, AUCi and peak reactivity.

### 3.3 Regression analyses predicting depressive symptom change from pregnancy to postpartum

The regression analyses examined the association between the CAR during pregnancy (n = 55) and changes in depressive symptoms two months postpartum (n = 43).

#### 3.3.1 Model 1: Area under the curve with respect to increase (AUCi)

In the unadjusted model, higher AUCi was significantly associated with a smaller reduction in depressive symptoms postpartum (β = 0.36, p = .02). In the adjusted model including the propensity score (gestational age, BMI, medication, country of birth, treatment allocation, smoking, and chronotype), this association remained significant (β = 0.36, p = .02), indicating that higher CAR was associated with less symptom improvement. The propensity score itself was not a significant predictor (β = −0.15, p = .30). See Fig. 1a.

**Fig. 1a:**
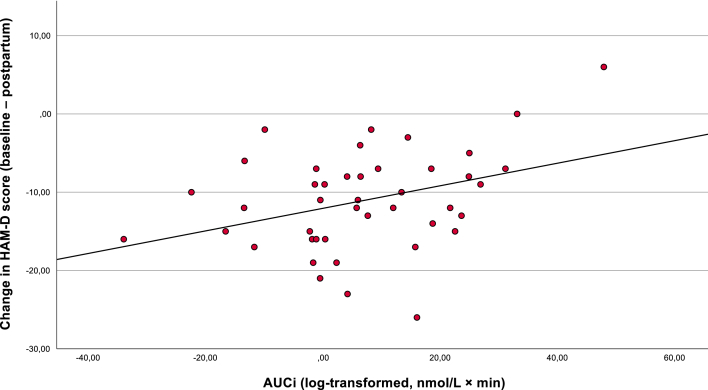
Association between cortisol AUCi during pregnancy and change in depressive symptoms postpartum (n = 43). The scatterplot shows the association between the cortisol awakening response measured as area under the curve with respect to increase (AUCi; log-transformed salivary cortisol levels) and the change in depressive symptoms from pregnancy (baseline) to two months postpartum. Change in HAM-D score was calculated as baseline minus postpartum score, with negative values indicating symptom improvement. Higher AUCi was significantly associated with less symptom reduction (β = 0.36, p = .02).

#### 3.3.2 Model 2: Area under the curve with respect to ground (AUCg)

In the unadjusted model, AUCg was not significantly associated with depressive symptom change (p = .19). In the adjusted model with the propensity score, this result remained non-significant (p = .28). Similarly, the propensity score was not significantly associated with depressive symptom change (β = 0.05, p = .71). See Fig. 1b.

**Fig. 1b:**
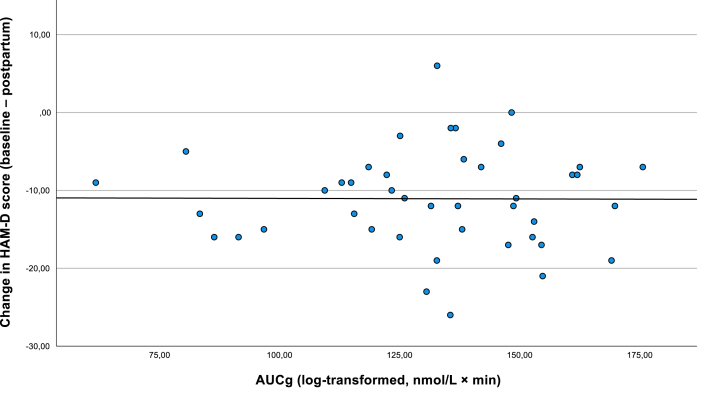
Association between total cortisol output (AUCg) during pregnancy and change in depressive symptoms postpartum (n = 43). The figure displays the relationship between AUCg (area under the curve with respect to ground; log-transformed cortisol) and the change in depressive symptoms (HAM-D score) from pregnancy to postpartum. No significant association was observed in the unadjusted model (β = 0.18, p = .19).

#### 3.3.3 Model 3: Peak cortisol reactivity

In the unadjusted model, higher peak cortisol reactivity was significantly associated with a smaller reduction in depressive symptoms postpartum (β = 0.326, p = .033).

In the adjusted model including the propensity score, this association remained significant (β = 0.324, p = .034), suggesting that greater peak reactivity predicted less symptom improvement. The propensity score itself was not a significant predictor (β = −0.17, p = .25). See Fig. 1c.

**Fig. 1c:**
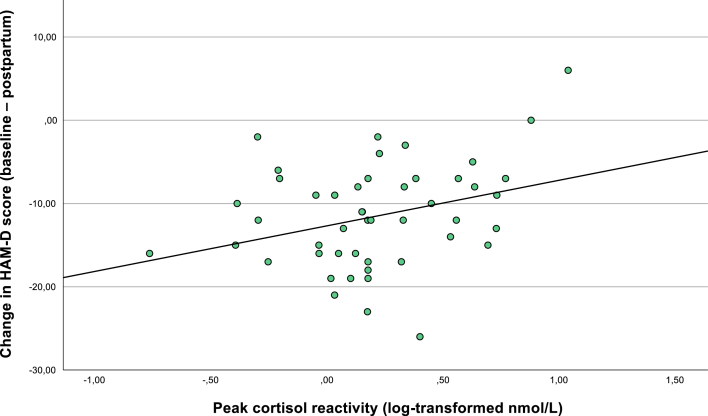
Association between peak cortisol reactivity during pregnancy and change in depressive symptoms postpartum (n = 43). The figure shows the association between peak cortisol reactivity - defined as the difference between log-transformed cortisol concentrations at 30 min post-awakening and awakening - and change in HAM-D scores from pregnancy to two months postpartum. Greater peak reactivity was associated with less symptom improvement in the unadjusted model (β = 0.33, p = .03).

All results are presented in Table 3.

**Table 3:** Regression analysis predicting change in depressive symptom trajectory from pregnancy to postpartum.

Model		B	SE	β	p-value
Step 1	CAR - AUCg	0.09	0.07	0.18	0.19
Step 2	CAR - AUCg	0.08	0.07	0.16	0.28
	Propensity score	0.08	0.22	0.05	0.71

Model 3		B	SE	β	p-value
Step 1	CAR - Peak reactivity	5.37	2.43	0.33	0.03∗
Step 2	CAR - Peak reactivity	5.34	2.42	0.32	0.03∗
	Propensity score	−0.10	0.08	−0.17	0.25

Note: The dependent variable is change in depressive symptoms (HAM-D score) from pregnancy (baseline) to postpartum (two months). Independent variables include different indices of the cortisol awakening response (CAR), i.e. area under the curve with respect to increase (AUCi, model 1); area under the curve with respect to ground (AUCg, model 2) and peak reactivity (change in cortisol value between awakening and +30 min after awakening; model 3). Coefficients are unstandardized beta values. SE = Standard error. p < .05. The model is adjusted using a propensity score, which accounts for gestational age, BMI, medication use (psychotropic and somatic), country of birth, treatment allocation (bright light condition vs. placebo), smoking status, and self-reported chronotype (morning, evening, or neutral preference).

## 4 Discussion

This study explores the relationship between the cortisol awakening response (CAR) during pregnancy and changes in depressive symptoms postpartum in women with depressive disorders. We found that higher CAR indices, specifically the area under the curve with respect to increase (AUCi) and peak reactivity, were significantly associated with a smaller reduction in depressive symptoms from pregnancy to two-months postpartum. In contrast, the area under the curve with respect to the ground (AUCg), a measure of total cortisol output, showed no significant association. These findings suggest that heightened cortisol reactivity may negatively impact recovery from depression in the peripartum period. The propensity score, accounting for multiple confounders (including treatment allocation, BMI and gestational age at baseline), did not significantly predict symptom change.

As the CAR reflects cortisol reactivity to a natural stressor (awakening), heightened reactivity may indicate an overactive or dysregulated hypothalamic-pituitary-adrenal (HPA) axis response. This exaggerated response could suggest increased stress reactivity or an impaired ability to regulate cortisol release following (perceived) stressors. Evidence suggests that the CAR plays a role in arousal, energy boosting, and anticipation, functioning as an adaptive mechanism to aid recovery from prior negative experiences and prepare for forthcoming demands [45]. For example, subjective feelings of loneliness, sadness, or threat before sleep have been linked to a greater CAR the following morning, while anticipation of daily challenges, such as work-related stress, has also been associated with elevated CAR [46,47].

In peripartum depression, a heightened CAR may indicate a maladaptive stress response, interfering with emotional recovery and mood stabilization. The link between heightened CAR and less symptom improvement suggests that HPA axis reactivity may serve as a marker for women at risk of persistent depressive symptoms, although research in larger samples and in populations with more severe depressive symptoms, is necessary to generalize the results. Differentiating between dynamic reactivity (AUCi, peak) and total cortisol output in the post-awakening period (AUCg), highlights the importance of addressing stress reactivity rather than overall cortisol levels. Screening for dysregulated CAR during pregnancy could guide targeted interventions, such as mindfulness or relaxation techniques [48].

Our results align with previous research demonstrating that elevated CAR is associated with heightened stress reactivity [28,49]. The observed relationship between higher AUCi and peak reactivity and a smaller reduction in depressive symptoms supports the hypothesis that an exaggerated stress response, may drive depressive symptom persistence [23]. In the peripartum period, these neurobiological disruptions are further amplified by pregnancy-specific hormonal shifts, including rising levels of placental corticotropin-releasing hormone (CRH) and estrogen withdrawal postpartum and a rapid decrease of CBG [5,50].

The peripartum period has a similarly high prevalence of depression as other life stages [51,52], despite the presence of unique hormonal changes, psychosocial stressors, and heightened cortisol reactivity. This suggests that while these factors may contribute to the onset and persistence of depressive symptoms, depression in other life stages is likely driven by different, but equally impactful, biological and psychosocial mechanisms. of these hormonal changes, psychosocial stressors, and heightened cortisol reactivity, which together could exacerbate onset and persistence of depressive symptoms.

As all women in our study were diagnosed with depressive disorder and we lacked a healthy control group for comparison, the findings could also suggest that differences in CAR reflect distinct underlying pathophysiological mechanisms of depressive symptoms in the peripartum period [53]. For instance, heightened CAR might indicate a hyperactive HPA axis, which is potentially linked to stress-related or anxious-depressive subtypes. In contrast, a blunted CAR, as observed in other contexts, could correspond to chronic, melancholic depression characterized by HPA axis “exhaustion” [54,55].

These findings highlight the potential value of CAR as a biomarker for identifying women at heightened risk of persistent depressive symptoms during the peripartum period. However, before CAR measurements can be integrated into clinical practice, further research in larger and more diverse samples is needed to validate these results and determining specific cut-off scores. Importantly, our findings suggest that stress reactivity, as captured by CAR, provides a more dynamic and sensitive marker of HPA-axis functioning compared to basal cortisol levels, which reflect more stable endocrine states rather than acute responsiveness to stressors. This aligns with previous research from our group, which found no association between peripartum depression and state-cortisol levels measured in hair [54], suggesting that chronic cortisol exposure alone may not sufficiently capture individual differences in stress vulnerability during pregnancy [56].

Further studies could investigate interactions between CAR and therapeutic interventions to determine whether the HPA axis responds to effective treatments or serves as a predictor of treatment outcomes. In this sample, no differences were found between bright light therapy and the control condition, either in CAR indices or symptom levels, as both groups showed significant improvement. This suggests that bright light therapy may not be an effective intervention for this population. Additionally, exploring the bidirectional relationship between CAR and depressive symptoms may clarify whether changes in stress reactivity are a cause or consequence of symptom improvement [12].

Future research may also examine whether specific symptom dimensions - such as affective versus neurovegetative features - differentially relate to CAR indices, potentially providing more refined insights into HPA axis involvement in peripartum depression.

### 4.1 Strengths and limitations

Strengths include the use of a well-characterized clinical sample of pregnant women with depressive disorders, a longitudinal design with availability of information on diverse potential confounders such as medication use, gestational age, and obstetric complications, and robust cortisol measurements (LC-MS/MS) [57]. Limitations include the absence of precise awakening times. Furthermore, BMI was measured during pregnancy rather than pre-pregnancy, which may be a less reliable indicator of baseline metabolic status and HPA-axis function [58]. While self-reported chronotype was collected and showed no significant association with CAR indices, future studies should incorporate precise awakening times using objective measures, such as actigraphy or sleep diaries, in combination with standardized wake-up procedures (e.g., alarm clocks). Another limitation is the relatively small sample size, which may limit generalizability. Another limitation was the potential for selection bias, as only participants with available salivary cortisol measurements at baseline were included. This may have led to systematic differences between included and excluded participants. However, we accounted for treatment allocation as a covariate in our analyses to mitigate its potential impact as participants were part of an RCT on bright light therapy [26]. Additionally, analyses adjusted for treatment allocation confirmed no differences in symptom trajectories between treatment arms.

## 5 Conclusion

This study underscores the relevance of heightened CAR indices during pregnancy, particularly AUCi and peak reactivity, in understanding the risk for persistent depressive symptoms during the peripartum period. Beyond identifying at-risk populations, future research should focus on whether CAR can serve as an individualized predictive marker for symptom trajectories, enabling more personalized risk stratification and early intervention.

## CRediT authorship contribution statement

**Carlinde W. Broeks:** Writing – review & editing, Writing – original draft, Methodology, Investigation, Formal analysis, Data curation, Conceptualization. **Babette Bais:** Writing – review & editing, Writing – original draft, Project administration, Methodology, Data curation, Conceptualization. **Rien Van:** Writing – review & editing, Writing – original draft. **Hilmar H. Bijma:** Writing – review & editing, Writing – original draft. **Elisabeth F.C. van Rossum:** Writing – review & editing, Writing – original draft. **Witte J.G. Hoogendijk:** Writing – review & editing, Writing – original draft, Conceptualization. **Mijke P. Lambregtse-Van den Berg:** Writing – review & editing, Writing – original draft, Supervision, Project administration, Funding acquisition, Data curation, Conceptualization. **Astrid M. Kamperman:** Writing – review & editing, Writing – original draft, Supervision, Project administration, Formal analysis, Conceptualization.

## Declaration of competing interest

The authors declare that they have no conflicts of interest relevant to this manuscript. No financial, personal, or institutional relationships could be perceived as influencing the research, analysis, or conclusions presented in this study.

All authors have contributed significantly to the work and have approved the final version of the manuscript for submission.
